# Plant Polyphenols and Their Anti-Cariogenic Properties:A Review

**DOI:** 10.3390/molecules16021486

**Published:** 2011-02-11

**Authors:** Gianmaria F. Ferrazzano, Ivana Amato, Aniello Ingenito, Armando Zarrelli, Gabriele Pinto, Antonino Pollio

**Affiliations:** 1 Department of Paediatric Dentistry, University of Naples “Federico II”, Naples, Italy; E-Mails: ivana.amato@libero.it (I.A.); ingenito@unina.it (A.I.); 2 Department of Organic and Biological Chemistry, University of Naples “Federico II”, Naples, Italy; E-Mail: zarrelli@unina.it (A.Z.); 3 Section of Plant Biology, Department of Biological Sciences, University of Naples “Federico II”, Napoli, Italy; E-Mails: gabpinto@unina.it (G.P.); anpollio@unina.it (A.P.)

**Keywords:** polyphenols, dental caries, anti-microbial action

## Abstract

Polyphenols constitute one of the most common groups of substances in plants. Polyphenolic compounds have been reported to have a wide range of biological activities, many of which are related to their conventional antioxidant action; however, increasing scientific knowledge has highlighted their potential activity in preventing oral disease, including the prevention of tooth decay. The aim of this review is to show the emerging findings on the anti-cariogenic properties of polyphenols, which have been obtained from several *in vitro* studies investigating the effects of these bioactive molecules against *Streptococcus mutans*, as well as *in vivo* studies. The analysis of the literature supports the anti-bacterial role of polyphenols on cariogenic streptococci, suggesting (1) a direct effect against *S. mutans*; (2) an interaction with microbial membrane proteins inhibiting the adherence of bacterial cells to the tooth surface; and (3) the inhibition of glucosyl transferase and amylase. However, more studies, particularly *in vivo* and *in situ*, are necessary to establish conclusive evidence for the effectiveness and the clinical applications of these compounds in the prevention of dental caries. It is essential to better determine the nature and distribution of these compounds in our diet and to identify which of the hundreds of existing polyphenols are likely to provide the greatest effects.

## 1. Introduction

Today, polyphenols occupy a unique place in science as the only class of bioactive natural products that the general public is aware of and has certainly heard about as a consequence of their presence in plant-derived foods and beverages and their inclusion in the formulations of well-marketed cosmetic [1,2,3] and parapharmaceutical products [4,5].

Polyphenols constitute one of the most common and widespread groups of substances in flowering plants, occurring in all vegetative organs, as well as in flowers and fruits. They are considered secondary metabolites involved in the chemical defence of plants against predators and in plant-plant interferences. Several thousand plant polyphenols are known, encompassing a wide variety of molecules that contain at least one aromatic ring with one or more hydroxyl groups in addition to other substituents. The biological properties of polyphenols include antioxidant [6], anticancer [7], andanti-inflammatory [8] effects.

Emerging findings suggest a variety of potential mechanisms of action by which polyphenols may prevent disease, such as the inhibition of bacterial replication enzymes, the induction of apoptosis in tumour cells, the stimulation of monocytes/macrophages to produce cytokines, and the stimulation of myeloperoxidase-dependent iodination of neutrophils [9]. The antimicrobial effects of polyphenols have also been widely reported as has their ability to inactivate bacterial toxins, and there is an increasing interest in this topic because plant polyphenols could represent a source of new anti-infective agents against antibiotic-resistant human pathogens. Today, dental caries are still one of the most common diseases in the world. The results of multi-variable modelling support the hypothesis that bacterial infection is important in the aetiology of dental caries [10]. The central role of the mutans streptococci in the initiation of caries on smooth surfaces and fissures of crowns of teeth suggests their role in the induction of root-surface caries [11]. This review presents the most important results on the anti-cariogenic properties of plant polyphenols in the light of the increasing scientific knowledge about the antimicrobial properties of these compounds.

### 1.1. Classification of Polyphenols

**Figure 1:**
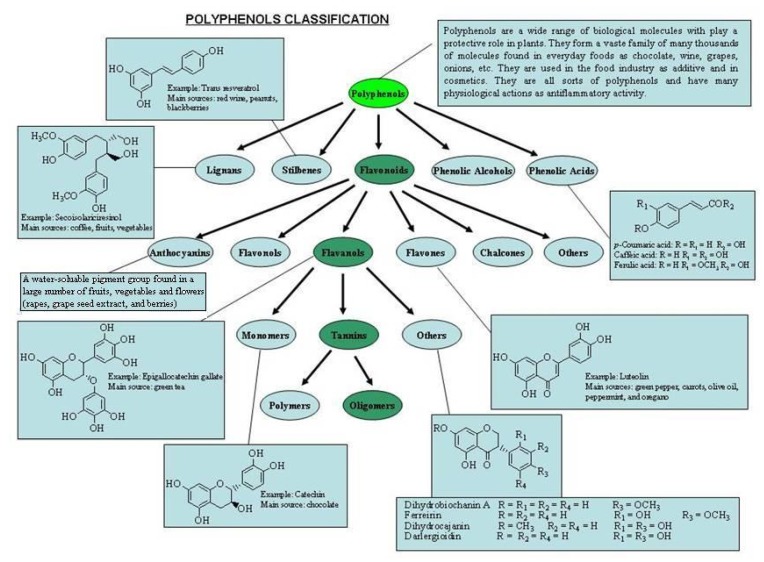
Polyphenol classification.

The empirical classification of plant polyphenols as molecules having a “tanning” action led to their being referred to in the early literature as “vegetable tannins”. Haslam [12] proposed the first comprehensive definition of the term “polyphenol”, attributing it exclusively to water-soluble phenolic compounds having molecular masses of 500 to 3,000–4,000 Da and possessing 12 to 16 phenolic hydroxyl groups and 5 to 7 aromatic rings per 1,000 Da. The original definition of “polyphenols” has broadened considerably over the years to include many much simpler phenolic structures (Figure 1). They encompass several classes of structurally-diverse entities that are essentially all biogenerated through either the shikimate/phenylpropanoid or the “polyketide” acetate/malonate secondary metabolic pathways [13], or both.

### 1.2. C_6_-C_3_ Phenylpropanoid Compounds

Some members of this huge class of natural products (>8,000 structures), usually bearing two mono- trihydroxyphenyl units, can serve as precursors to oligo- and polymeric phenolic systems. The general phenylpropanoid metabolism furnishes a series of hydroxycinnamic acids (C_6_-C_3_) differing from one another by the number of hydroxy and methoxy groups on their phenyl units (*i.e.*, ferulic acid, caffeic acid). These monophenolic carboxylic acids are often found esterified to polyols. Through hydration, esterification, and phenolic oxidative coupling reactions, caffeic acid also gives rise to oligomeric structures.

### 1.3. C_6_-C_2_-C_6_ Polyhydroxystilbenes

The phenylpropanoid/acetate hybrid metabolic pathway leads to another important class of phenolic substances, the polyhydroxystilbenes (C_6_-C_2_-C_6_). The most famous example of this class is the phytoalexin *trans*-resveratrol (*i.e.*, 3,5,4’-trihydroxy-*trans*-stilbene), which has been the centre of much scientific attention and media exposure following its biological evaluation as a cancer chemopreventative and its detection in red wine [14,15,16]. Such phenolic systems featuring a conjugated carbon-carbon bond in their side-chains are particularly prone to undergo oligomerisation events via coupling of delocalised phenoxy radicals generated by one-electron oxidation reactions.

### 1.4. Lignin Derivatives

Much like the hydroxycinnamic acids, esters and alcohols that are converted into lignan/neolignan dimers (C_6_-C_3_)_2_ and plant cell wall lignin polymers [(C_6_-C_3_)_n_] by such oxidative coupling processes, resveratrol and its hydroxystilbenoid analogues can react in the same manner to furnish polyphenolic oligomers. The presence of more than one hydroxyl group on a benzene ring or other arene systems does not make them “polyphenols”. Catechol, resorcinol, and pyrogallol are all di- and trihydroxylated benzene (C_6_) derivatives, but they are still defined as “polyphenols” according to the IUPAC official nomenclature rules of chemical compounds. Many monophenolics are often called “polyphenols” by the cosmetic and parapharmaceutical industries, but they cannot be classified as such by any scientifically accepted definition. The meaning of the chemical term “phenol” includes both the arene ring and its hydroxyl substituent(s), and the term “polyphenol” should be confined, in a strict chemical sense, to structures bearing at least two phenolic moieties, independently of the number of hydroxyl groups that they each bear. Moreover, many natural products of various biosynthetic origins do not contain more than one phenolic unit. It is, for example, the case for many alkaloids derived from the amino acids phenylalanine and tyrosine. The term “polyphenol” should be used to define compounds exclusively derived from the shikimate/phenylpropanoid and/or the polyketide pathways, featuring more than one phenolic unit and deprived of nitrogen-based functions.

### 1.5. Categories of Polyphenols

Polyphenols can be classified into several categories: The *flavonoids* are obtained by the lengthening of the side chain of cinnamic acids by the addition of one or more C_2_ units, typically resulting in mixed biosynthesis metabolites with important biological properties. In particular, these polyphenolic compounds have 15-carbon skeletons, represented as the C_6_-C_3_-C_6_ system. The flavonoids are 1,3-diarylpropanes, isoflavonoids are 1,2-diarylpropanes, and neoflavonoids are1,1-diarylpropanes. The term “flavonoid” was first used by Geismann and Hinreiner [17] in 1952 for the classification of those compounds whose structure is correlated to the 2-phenylchroman heterocyclic system (flavan). Their skeleton is made up of two benzene rings with a chain of three carbon atoms of a γ-pyrone system. Thus, the several flavonoidic compound classes differ in the oxidation states of their heterocyclic systems. Single constituent flavonoids of every class are mainly distinguished by the number and the stereochemistry of the hydroxyl groups and/or methoxyls on the two benzene rings and/or the heterocyclic system. These replacements are found in defined positions of flavonoids, such that they indicate a different biogenetic origin for two aromatic rings, A and B. In many cases, then, the flavonoidic compounds have been isolated, such as glycosides, one or more hydroxyl groups are joined with a hemiacetalic bound, generally through the C-1 carbon and with a bond of type *β*, to one or more sugars. Flavonoids are fundamentally important for ecological role as pigment in flowers and fruits. Flavonoids are important for plants' ecological roles in that they are the pigments that give colour to fruits and flowers, thereby attracting pollinators. The *coumarins* are typical metabolites of higher plants. The benzo-2-pyrone nucleus of the simple coumarins derives from the phenylacrylic skeleton of cinnamic acids via *orto*-hydroxylation, *trans*-*cis* isomerisation of the side chain double bond, and lactonisation. The sequences and the mechanisms of such processes are still uncertain in most cases, in particular *trans*-*cis* isomerisation of the double bond could occur under enzymatic catalysis, through a photochemical process, or through other mechanisms, such as a reduction-dehydrogenation sequence. *The lignans* comprise a group of natural compounds with carbon skeletons derived from two phenylpropane units joined together by at least one carbon-carbon bond between the two central *β*-carbons of the C_3_ chains (lignans) or by bonds other than the *ββ*’-carbon-carbon bond (neolignans). The aromatic rings are usually oxysubstituted, particularly at the *para* position with respect to side-chain substitution. No lignan has been isolated with an unsubstituted phenyl ring and monosubstituted examples are also rare. Generally at least one of the aromatic rings is oxygenated at the 3- and 4-positions. In some cases one of the aromatic rings is modified partially or completely to an alicyclic system which may also undergo cyclization process with the side chain of other C6-C3 units [18,19].

Compounds formed by shortening of the side chain of the phenylpropane skeleton can be divided into three groups: the *C_6_-C_2_ compounds*, with loss of the carboxylic carbon, to form alcohols or catabolites of cinnamic acids [18,20,21] used by plants for example in the biosynthes is of alkaloids, the *C_6_-C_1_ compounds*, such as benzoic acids and their variously oxygenated derivatives are very common in Nature [18,20,21] and they are usually found as glycosides that is conjugated with an aldose (usually D-glucose) through phenolic hydroxyls, or as esters, that is with their carboxylic group esterified with either alcohols or polyphenols. Finally, the *C_6_* compounds, derive from the non-oxidative decarboxylation of the corresponding benzoic acids to form hydroquinones which are rarely found in higher plants [22].

## 2. Antibacterial Activity of Plant Polyphenols

Phenolic compounds have diverse defensive functions in plants, such as cell wall strengthening and repair (lignin and suberin) [23] and antimicrobial and antifungal activities. Some polyphenols are phytoanticipins, compounds with a defensive role that are not synthesised in response to a pathogen attack but rather are constitutively present in plant cells [24]. Phenolic constituents occur on the surface of plants or in the cytoplasmic fraction of the epidermal cells, where they act as a deterrent to pathogens. In contrast, phenolic phytoalexins are secreted by wounded plants or in response to incompatible pathogens [25]. The induced defence response includes cell death and the formation of a lesion that limits the growth of the pathogen. Cells around the lesion accumulate polyphenols and other antibiotic compounds [26]. Pholyphenols as catechin act on different bacterial strains belonging to different species (*Escherichia coli*, *Bordetella bronchiseptica*, *Serratia marcescens*, *Klebsiella pneumonie*, *Salmonella choleraesis*, *Pseudomonas aeruginosa*, *Staphilococcus aureus*, and *Bacillus subtilis*) by generating hydrogen peroxide [27] and by altering the permeability of the microbial membrane [28]. Microbes stressed by exposure to polyphenols upregulate proteins related to defensive mechanisms, which protect cells while simultaneously downregulating various metabolic and biosynthetic proteins involved, for example, in amino acid and protein synthesis as well as phospholipid, carbon, and energy metabolism [29]. Moreover, polyphenols have been reported to interfere with bacterial quorum sensing, *i.e.*, the production of small signal molecules by bacterial cells of *Escherichia coli*, *Pseudomonas putida* and *Burkholderia cepacia* that trigger the exponential growth of a bacterial population [30].

A large body of evidence indicates that many plants used as folk remedies contain high concentrations of polyphenolic compounds [31]. Plants from a wide range of angiosperm families show antibacterial activity. In one study, 35 of 146 seed extracts inhibited microbial growth, and the biocidal activity of the seed extracts correlated with their polyphenol content. Plants from more than 20 different families, including Asteraceae, Fabaceae, Poaceae, Lythraceae, Onagraceae, Polygonaceae, Primulaceae, and Verbenaceae showed bactericidal action [32]. Members of the Geraniaceae and Rosaceae families are also rich in polyphenolic compounds with antimicrobial activity [33], and *Cydonia oblonga* Miller, a member of the latter family, was found to be an important source of polyphenols that are active against bacteria growth [34]. Polyphenols with relevant biocidal activity have been isolated from members of other plant families: Taguri *et al.* [35] isolated castalagin and protodelphinidin flavenoids that are fundamentally important for ecological role as pigments in flowers and fruits, from *Castanea crenata* Siebold & Zucc (Fagaceae) and *Elaeocarpus sylvestris* (Lour.) Poir. var. *ellipticus* (Elaeocarpaceae), respectively, and found them to be effective against different bacterial strains [35]*.*

## 3. Pathogenesis of Dental Caries

Dental caries is a multi-factorial infectious disease, arising from the interplay between oral flora, the teeth and dietary factors. Dietary carbohydrates, mainly mono- and disaccharides, are absorbed into dental plaque and broken down into organic acids by the micro-organisms present in dense concentrations. The mineral content of teeth is sensitive to increases in acidity from the production of lactic acid. Specifically, a tooth (which is primarily mineral in content) is in a constant state of back-and-forth demineralization and remineralization between the tooth and surrounding saliva. When the pH at the surface of the tooth drops below 5.5, demineralization proceeds faster than remineralization (meaning that there is a net loss of mineral structure on the tooth's surface). This results in the ensuing decay.

Several strains of oral streptococci are capable of initiating the formation of dental plaque, which plays an important role in the development of caries and also of periodontal disease in humans [36]. Dental plaque has been implicated as an important etiologic factor in dental caries [37]. It is a complex bacterial biofilm community for which the composition is governed by factors such as cell adherence, coaggregation, and growth and survival in the environment [38]. Plaque bacteria utilize the readily fermentable carbohydrates on tooth surfaces to produce acids that promote and prolong the cariogenic challenge to teeth, leading to enamel demineralization and tooth decay. The development and progression of dental caries depends on the amount of food particles that become trapped on the surfaces of teeth that may serve as ready sources of fermentable carbohydrates, thereby promoting acid production by plaque bacteria. This prolongs the cariogenic challenge to the teeth, leading to enamel demineralization and tooth decay.

The major aetiological players are thought to be the two α-haemolytic streptococci, *Streptococcus mutans* and *Streptococcus sobrinus*, which are potent cariogenics, although several other types of bacteria (notably lactobacilli and actinomyces) may also be involved.

*S. mutans* produces three types of glucosyltransferase (GTFB, GTFC, and GTFD) which polymerize the glucosyl moiety from sucrose and starch carbohydrates into α1,3- and α1,6-linked glucans [39,40]. Binding to glucans by glucan binding proteins (GbpA, -B, -C and -D) and by the Gtfs facilitates bacterial adherence to tooth surfaces, inter-bacterial adhesion and accumulation ofbiofilms [40,41]. GtfBC&D and GbpABC&D, together with the adhesive extracellular glucans, constitute the sucrose-dependent pathway for *S. mutans* to establish on tooth surface and are of central importance in plaque formation and development of caries [39,40].

The adherent glucan also contributes to the formation of dental plaque, in which the accumulation of acids leads to localised decalcification of the enamel surface. The carbohydrate substrates can become available either directly (as sugar ingested in food or drink) or be derived from dietary starch by the action of bacterial or salivary amylases, or both. Polyphenols have been shown in many studies, both in animals and in humans, to interfere specifically with each of the processes described [42].

## 4. Anti-Cariogenic Action of Polyphenols

A variety of compounds capable of controlling dental caries have been extensively surveyed; however, only limited numbers of compounds from natural products are available because of effectiveness, stability, odour, taste, and economic feasibility [43,44]. The effects of polyphenols have been surveyed through both *in vitro* studies investigating the effect of polyphenols against mutans streptococci and *in vivo* studies in animals and humans [45,46,47,48].

### 4.1. *In Vitro* Studies

**Figure 2:**
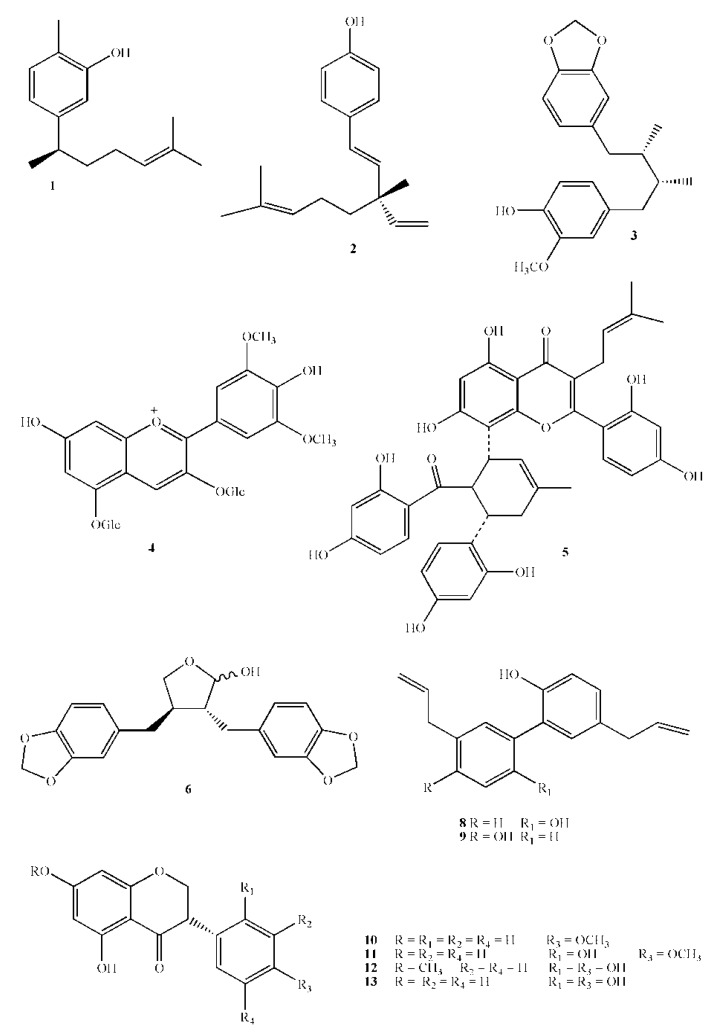
Chemical structures of active polyphenols. **1** Xanthorrhizol; **2** Bakuchiol; **3** Macelignan; **4** Malvin; **5** Kuwanon G; **6** (−)-Cubebin; **8** Magnolol; **9** Honokiol; **10** Dihydrobiochanin A; **11** Ferreirin; **12** Dihydrocajanin; **13** Dalbergioidin.

Studies on the activities of phenolic compounds toward cariogenic bacteria can be divided based on the chemical structure of the compound under study (Figure 2–Figure 4 and Table 1). Few studies deal with the anti-streptococcal action of simple polyphenols. Xanthorrhizol (XTZ), isolated from *Curcuma xanthorrhiza* Roxb., has been reported to possess antibacterial activity against several oral pathogens, and it has shown to have rapid bactericidal activity against *S. mutans* [49]. The activity of XTZ in removing *S. mutans* biofilm was dependent on its concentration and exposure time as well as the growth phase of the biofilm. A concentration of 5 µmol L^−1^ of XTZ completely inhibited biofilm formation by *S. mutans* at the adherent phase of growth, whereas 50 µmol L^−1^ of XTZ removed 76% of the biofilm at the plateau accumulated phase after a 60-min exposure. Another simple phenol, bakuchiol, isolated from *Psoralea corylifolia* L, showed inhibitory activity against *S. mutans* [50]*.* Yanti *et al.* [51] reported anti-biofilm activity of macelignan, isolated by nutmeg (*Myrisica fragrans* Houtt.) against oral bacteria including *S. mutans*, *S. sanguinis* and *Actinomyces viscosus*. This study demonstrated that macelignan activity at 10 µg/mL for a 30 min exposure time could remove more than half of each single oral biofilm formed by *S. mutans*, *S. sanguinis* and *A. viscosus* at the plateau accumulated phase (24 h).

From an ethanol extract of *Alcea longipedicellata* (Malvaceae) malvidin-3,5-diglucoside (malvin) was identified as the principal constituent which was responsible for antibacterial activity. 0.1% malvin could inhibit strongly acid producing ability of *S. mutans* and was about 60% effective in inhibiting bacterial adherence [52]. Kuwanon G, isolated from a methanol extract of root bark of *Morus alba* L. showed bactericidial action in 1 min. at a concentration of 20 μg/mL against *S. mutans* and other cariogenic bacteria as *S. sobrinus*, *S. sanguinis* and *Porpyromonas gengivalis* [53].

The activity of crude ethanol extract from *Piper cubeba* seeds, the purified compounds (−)-cubebin and its semi-synthetic derivatives were evaluated against oral pathogens. The crude ethanol extract was more active against *S. salivarium* (MIC value of 80 μg/mL) and purified compounds and semisynthetic derivaties displayed MIC values ranging from 0.20 mM for *S. mitis* to 0.32 mM for *S. mutans* [54].

The active flavonoid compound, quercetin-3-*O*-α-L-arabino-pyranoside (guaijaverin) isolated from *Psidium guajava* L. demonstrated high potential antiplaque agent by inhibiting the growth of the *S. mutans* [55]. Magnolol and honokiol isolated from extracts of *Magnolia* sp. bark have a phenyl-propanoid dimer structure and are active against the cariogenic bacterium *S. mutans* (M.I.C. 6.3 mg/mL) [56].

**Figure 3:**
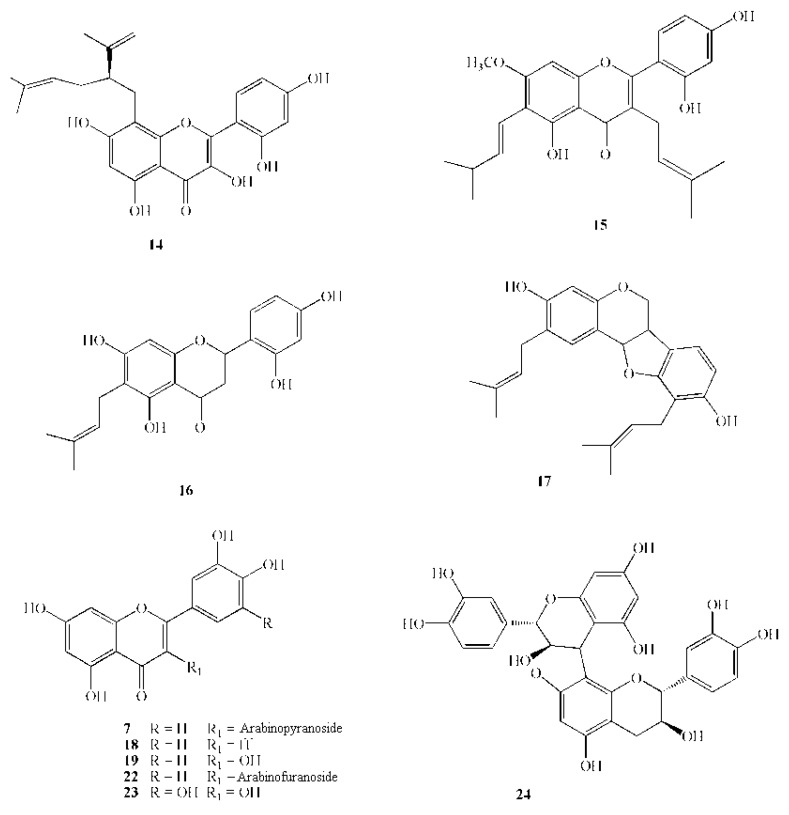
Chemical structures of active polyphenols: **7** Guaijaverin; **14** Lavandulylflavanone; **15** Artocarpin; **16** Artocarpesin; **17** Erycristagallin; **18** Luteolin; **19** Quercetin; **22** Quercetin-3-arabinofuranoside; **23** Myricetin.

**Figure 4:**
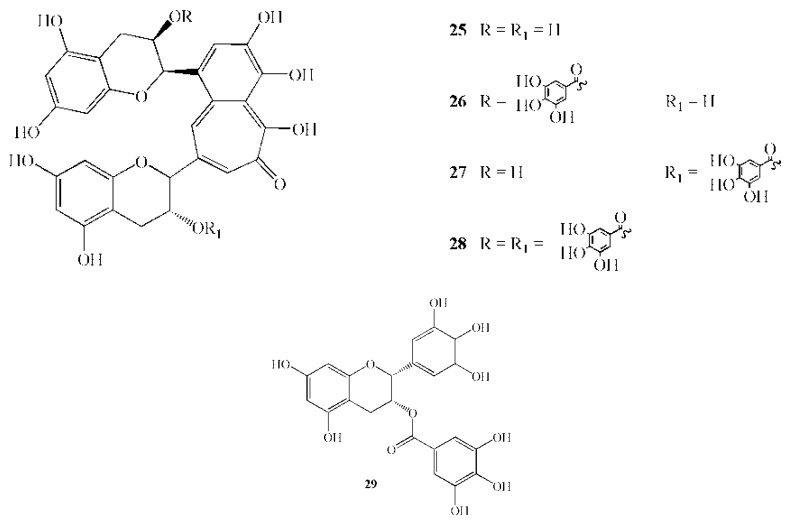
Chemical structures of active polyphenols: **25** Theaflavin; **26** Theaphlavin monogallate A; **27** Theaphlavin monogallate B; **28** Theaphlavin digallate; **29** Epigallocathechin gallate.

**Table 1:** Activity of plant phenolics against *Streptococcus mutans*.

N.	Name	Mol. weight	Plant name	Part of the plant	Activity against *S. mutans*	References
**1**	Xanthorrhizol	218.3	*Curcuma xanthorrihiza* Roxb	rhizome	5 mmol L^−1^ inhibit biofilm formation	[49]
**2**	Bakuchiol	256.4	*Psoralea corylifolia* L.	seeds	20 µg/mL prevented growth	[50]
**3**	Macelignan	328.4	*Myristica fragrans* Houtt*.*	seeds	10 μ/mL for 30’ exposure removed >50% of primary biofilm formed by *S. mutans*, *S. sanguinis* and *A . viscosus*	[51]
**4**	Malvin	655.2	*Alcea longipedicellata* I. Riedl	flowers	M.I.C. 0.16 mg/mL for *S. mutans*	[52]
**5**	Kuwanon G	692.7	*Morus alba* L*.*	Root bark	M.I.C. 8 μg/mL	[53]
**6**	(−)-Cubebin	356.4	*Piper cubeba* L.	seeds	M.I.C. 0.32 mM	[54]
**7**	Guaijaverin		*Psidium guaiava L.*	leaves	M.I.C. 4 mg/mL	[55]
**8**	Magnolol	266.3	*Magnolia officinalis*	bark	0.32 mg/mL reduced by 87.3% GTF activity	[56]
**9**	Honokiol	266.3	*Magnolia officinalis*	bark	0.32 mg/mL reduced by 58.1% GTF activity	[56]
**10**	Dihydrobiochanin A	286.3	*Swartzia polyphylla* DC	heartwood	M.I.C. 50 µg/mL	[65]
**11**	Ferreirin	302.3	*Swartzia polyphylla* DC	heartwood	M.I.C. 50 µg/mL	[65]
**12**	Dihydrocajanin	302.3	*Swartzia polyphylla* DC	heartwood	M.I.C. 100 µg/mL	[65]
**13**	Dalbergioidin	288.3	*Swartzia polyphylla* DC	heartwood	M.I.C. 100 µg/mL	[65]
**14**	Lavandulylflavanone	438.5	*Sophora exigua* Craigg	heartwood	Growth inhibition in the range 1.56–6.25 µg/mL	[66]
**15**	Artocarpin	436.5	*Artocarpus heterophyllus* Lam.	heartwood	M.I.C. 6.25 µg/mL	[67]
**16**	Artocarpesin	354.4	*Artocarpus heterophyllus* Lam.	heartwood	M.I.C. 6.25 µg/mL	[67]
**17**	Erycristagallin	392.5	*Erythrina variegata* L.	root	M.I.C. 6.25 µg/mL	[68]
**18**	Luteolin	286.2	*Perilla frutescens* Britton var. *japonica* Hara.	seeds	M.I.C. 50–100 µg/mL (on different *S. mutans* strains)	[69]
**19**	Quercetin	302.2	Commercial source	-	Inhibition of adhesive glucan format ion in the range 1.5–50 µg/mL	[73]
**20**	Proanthocyanidins	/	*Humulus lupulus* L.	bracts	0.01%, Hop Bracts Polyphenols (HBP) containing 35% proanthocyanidins caused 80% inhibition of GTF	[79]
**21**	Tannins	/	*Areca catechu* L.	nut	50% of growth inhibition at a 15% concentration	[82]
**22**	Quercetin-3-arabinofuranoside	434.3	*Vaccinium macrocarpon* Ait.	fruit	21–41% Inhibition of GTF activity at500 mmol L^−1^	[82]
**23**	Myricetin	318.0	*Vaccinium macrocarpon* Ait.	fruit	15-28% Inhibition of GTF activity at500 mmol L^−1^	[82]
**24**	Procyanidin A2	576.1	*Vaccinium macrocarpon* Ait.	fruit	21–41% Inhibition of GTF activity at500 mmol L^−1^	[82]
**25**	Theaflavin	564.1	*Camellia sinensis* L.	leaves	Inhibition of GTF activity in the range 1–10 mM	[85]
**26**	Theaphlavin monogallate A	716.3	*Camellia sinensis* L.	leaves	Inhibition of GTF activity in the range 1–10 mM	[85]
**27**	Theaphlavin monogallate B	716.3	*Camellia sinensis* L.	leaves	Inhibition of GTF activity in the range 1–10 mM	[85]
**28**	Theaphlavin digallate	868.1	*Camellia sinensis* L.	leaves	Inhibition of GTF activity in the range 1–10 mM	[85]
**29**	Epigallocathechin gallate	458.4	*Camellia sinensis* L.	leaves	167 mg/L caused 91% growth inhibition*	[85]

M.I.C. = Minimum Inhibition Concentration. GTF = Glucosyltransferases.

There is a large body of evidence supporting the inhibition of cariogenic bacteria by larger phenolic compounds, which are considered the “true” polyphenols. Research on this subject can be divided into two groups: (a) studies on fractions of plant extracts containing high concentrations of polyphenols, without the identification of individual compounds occurring in the extracts and (b) reports of the antibacterial activity of specific polyphenols.

The first group includes some early studies, such as that performed by Ooshima who examined the inhibitory effects of the cacao bean husk extract (CBH) on the caries-inducing properties of mutans streptococci *in vitro* and on caries development in specific pathogen-free Sprague-Dawley rats infected with mutans streptococci. He demonstrated that the CBH reduced the growth rate of almost all oral streptococci examined, which resulted in the reduction of acid production [57].

Subsequently, phenolic substances were suggested to be responsible for the observed anti-caries effect of cocoa powder [58], probably due to their inhibition of the synthesis of water-insoluble glucans [59].

Onion extracts have been reported to act on *Streptococcus mutans* and *Streptococcus sobrinus* as well as *Porphyromonas gingivalis* and *Prevotella intermedia*, which are considered to be the main causal bacteria of adult periodontitis [60]. Although no active components of the onion extracts were identified, onion is among the richest sources of flavonoids and contributes significantly to the overall dietary intake of flavonoids [61].

An *in vitro* study demonstrated that the tea polyphenol (TP) has no effect on de/remineralisation of enamel blocks, but it exerts an anti-caries effect via an anti-microbial mode-of-action [62].Smullen *et al.* [63] have shown that extracts from unfermented cocoa, green tea, and red grape seeds, all with a high polyphenol content, are effective against *S. mutans* and reduce its adherence to glass. Moreover, grape seed extracts inhibit the growth of anaerobic bacteria, such as *Porphyromonas gingivalis* and *Fusobacterium nucleatum*, associated with periodontal diseases [64].

There are numerous reports of the anti-streptococcal action of flavonoids. Three known isoflavanones, dihydrobiochanin A, ferreirin and darlbergioidin, and one new isoflavanone,5,2',4'-trihydroxy-7-methoxyisoflavanone (dihydrocajanin), which was isolated from *Swartzia polyphylla* DC heartwood, had potent activity against cariogenic bacteria [65]. A lavandulylflavone isolated from *Sophora exigua* Craig completely inhibited the growth of oral bacteria, including primary cariogenic mutans streptococci, other oral streptococci, actinomycetes, and lactobacilli, at concentrations of 1.56 to 6.25 mg/mL [66]. Isoprenylflavones from *Artocarpus heterophyllus* showed antibacterial activity against cariogenic bacteria [67]. Sato *et al.* [68] reported that erycristagallin from *Erythrina variegata* showed a high antibacterial activity against mutans streptococci, other oral streptococci, actinomycetes, and lactobacilli.

In recent years, polyphenols from some edible plants have attracted attention as potential sources of agents capable of controlling the growth of oral bacteria. Extracts from *Perilla frutescens* var. *japonica* seeds have shown inhibitory activity against oral cariogenic Streptococci and periodontopathic *Porphyromonas gingivalis*. *Perilla* seed polyphenols were isolated and their activity was tested. The flavonoid luteolin was the phenol that was most active against bacterial growth [69].

Sunphenon is a mixture of flavonols isolated from leaves of *Camellia sinensis*. The major components of this mixture are (+)-catechin, (+)-gallocathechin, (−)-epicathechin, (−)-epicathechin gallate, (−)-epigallocathechin, and (−)-epigallocathechin gallate [70]. The addition of Sunphenon to *S. mutans* JC-2 (c) decreased cell viability; multiple applications of Sunphenon caused the death of cells, and the maximum effect was seen with treatment of 60 and 90 minutes. [71].

### 4.2. Inhibition of Adherence

The adherence of bacterial cells to the tooth surface is of great importance to the development of carious lesions, and interference with some of the mechanisms of adherence can prevent the formation of carious lesions [72]. Polyphenols are able to interact with microbial membrane proteins, enzymes, and lipids, thereby altering cell permeability and permitting the loss of protons, ions, and macromolecules [28]. One of the first studies on this topic reported that quercetin, in the range 12.5–50 mg/mL, was effective in preventing adhesive glucan formation by *S. mutans* strains [73]

A chromatographically isolated oolong tea polyphenol (OTF6) may inhibit bacterial adherence to the tooth surface by reducing the hydrophobicity of mutans streptococci [61]. An *in vitro* study demonstrated that when *S. mutans* JC-2 (c) was pretreated with Sunphenon, its cellular attachment to a saliva-treated hydroxyapatite surface was significantly reduced [71].

Barley coffee (BC) interferes with *Streptococcus mutans* adsorption to hydroxyapatite. A low-molecular-mass (<1,000 Da) fraction containing polyphenols, zinc, and fluoride ions and a high-molecular-mass (>1,000 kDa) melanoidin fraction displayed strong anti-adhesive properties towards *S. mutans* [74]. A cocoa polyphenol pentamer (the most active component from M.I.C. studies) significantly reduced biofilm formation and acid production by *S. mutans* and *S. sanguinis*. [75].

### 4.3. Inhibition of Glucosyltransferase and Amylase

The enzymatic activity of glucosyl transferase from *Streptococcus mutans* is inhibited by plant polyphenols. Apple polyphenols extracted from immature fruits markedly reduced the synthesis of water-soluble glucans by glycosyl transferases (GTF) of *S. mutans* and *S. sobrinus* but did not inhibit salivary α-amylase activity. GTF inhibitors from apples are high-molecular-weight polyphenols with a chemical structure similar to catechin-based oligomeric forms and/or gallate-ester compounds [76]. Procyanidins from betel nuts (the seed of *Areca catechu* L.) were the major inhibitors of glucosyltransferase from *S. mutans* [77]. A high-molecular-weight polyphenol of *Humulus lupulus* L. (HBP) inhibited the cellular adherence of *S. mutans* MT8148 (serotype C) and *S. sobrinus* ATCC 33478 (serotype g) at much lower concentrations than those needed for the polyphenols extracted from oolong tea or green tea leaves. Furthermore, HBP also inhibited the action of glucosyltransferase, which was involved in the synthesis of water-insoluble glucan, but did not suppress the growth or acid production of the bacteria [78]. *H. lupulus* polyphenols significantly reduced the growth of *S. mutans* compared to the control. After an 18-hour incubation, HBP at 0.1% and 0.5% significantly reduced lactic acid production, and HBP at 0.01%, 0.1%, and 0.5% also suppressed water-insoluble glucan production [79]. The polyphenols from bracts of *H. lupulus* were purified by countercurrent chromatography (CCC). The most potent cavity-prevention activity was found in a very hydrophilic fraction, whose major components were high-molecular-weight substances, probably proantho-cyanidins, consisting of approximately 22 catech in units in their structures [80].

Grape and pomace phenolic extracts inhibited GTF of *S. mutans* at concentrations of 62.5 µL/mL. These extracts had qualitative and quantitative differences in their phenolic content but similar activity toward *S. mutans* GTF [81]. Extracts of flavonols (FLAV) and proanthocyanidins (PAC) from American cranberry (*Vaccinium macrocarpon* Ait.), alone or in combination, inhibited the surface-adsorbed glucosyltransferase and F-ATPase activities as well as acid production by *S. mutans* cells [82]. Flavonols and proanthocyanidins moderately inhibited the activity of surface-adsorbed GTF and disrupted acid production by *S. mutans* cells without killing them. The combination of three flavonoids, quercetin-3-arabinofuranoside, myricetin, and procyanidin, displayed pronounced biological effects on *S. mutans*, suggesting that the bactericidal activity could be the result of synergistic effects of flavonoids occurring in cranberry extracts [83]. A subsequent study by Yarnanaka-Omada *et al.* has confirmed that cranberry polyphenols are effective against hydrophobicity, biofilm formation, and bacterial growth of *S. mutans* [84].

Extracts of oolong tea and its chromatographically isolated polyphenolic compound inhibited insoluble glucan synthesis from sucrose by the GTases of *Streptococcus mutans* MT8148R and *S. sobrinus* 6715 [85]. Moreover, both extracts caused a decrease in the cell-surface hydrophobicity and aggregation of *S. mutans*, *S. oralis*, S. *sanguinis*, and *S. gordonii* [86]. Among the flavonoids isolated from tea infusions, theaflavin and its mono- and digallates were strong inhibitors of the synthesis of adherent water-insoluble glucans from sucrose catalysed by a glucosyltrasferase (GTF); (+)-catechin, (−)-epicatechin, and their enantiomers were moderately active, and galloyl esters of (−)-epicatechin, (−)-epigallocatechin, and (−)-gallocatechin showed increased inhibitory activities [87].

### 4.4. *In Vivo* Studies

Research in the field of dental caries using human subjects has been restricted for a number of reasons. First, dental decay is a disease of slow progression. Indeed, it has been estimated that a new lesion in a permanent tooth takes between 18 and 60 months to become clinically detectable [88]***.***Second, once established, a lesion is irreversible, thus experimental induction of caries is wholly unethical. Third, because of the length of the study period, it is quite impossible to obtain dietary histories and even less possible to control dietary intake. Fourth, perhaps most importantly, diet is but one of a large group of secondary factors, many of which may still be unknown, that contribute to an individual’s experience of this multifactorial disease.

For these reasons, most of the research on dental caries and diet has been carried out in animals, the rat model being by far the most common. Because of the dental and other obvious differences between humans and rats, the application of these animal findings to humans must be carried out with great caution. Clearly, this problem has greatly restricted the rate of progress in our knowledge and understanding of the precise role of dietary factors in relation to dental decay.

The majority of current commercial antiplaque products are antimicrobial compounds, but many antibiotic and chemical bactericides currently used to prevent bacterial infection disturb the bacterial flora of the oral cavity and digestive tract [89]. According to Eley [90], commercial mouthwashes can be grouped in three categories:

(1) Mouthwashes with good substantivity and antibacterial spectrum with a good anti-plaque effects. To this group belong biguanides as chlorhexidine; the effect of concentrated 1% chlorhexidine gel, on oral bacteria salivary levels can be observed after a couple of applications but this use requires professional supervision [91];
(2) Mouthwashes agents with little or no substantivity but with a good antibacterial spectrum. They have plaque inhibitory effects but lack true anti-plaque effects. In this category are included: cetyl pyridinium chloride, a quaternary ammonium compound, Listerine, which contains essential oil and phenolics (menthol, thymol, and eucalyptol), and triclosan, a trichlora-2'-hydroxydiphenyl ether;
(3) Antiseptic mouthwashes that have be shown to have antibacterial effects *in vitro* but in clinical studies have been shown to have low/negligible plaque inhibitory effects. Hexetidine (Oraldene), povidone iodine, oxygenating agents and the natural product sanguinarine, a benzophenanthridine alkaloid, are members of this third group.

Presently, no polyphenol has been included in the formulation of mothwashes or toothpaste. An eligible polyphenol should combine oral retentiveness with antibacterial activity, thus maintaining a prolonged activity in the mouth. However, over the last decade the protective effects of polyphenols was instigated also in some human studies.

The administration of oolong tea extract and the isolated polyphenol compound in the diet and drinking water resulted in significant reductions in caries development and plaque accumulation in the rats infected with mutans streptococci [85]. A study on black tea has determined the effects of a standardised black tea extract (BTE) on caries formation in inbred hamsters that were fed regular and cariogenic diets. The frequent intake of black tea significantly decreased caries formation by 56,6% in hamster on a regular diet and by 63,7% in hamsters on a cariogenic diet [92].

A clinical test to evaluate the effect of a mouthwash containing 0.1% *H*. *lupulus* bract polyphenols (HBP) on dental plaque regrowth over three days has shown that the HBP mouthwash was effective in reducing dental plaque regrowth (total plaque reduction of 25.4% compared with the placebo), and it lowered the number of mutans streptococci [93].

Furthermore, on human, significantly lower mean Plaque Index was observed among 35 volunteers who rinse their mouth with oolong tea extract OTE solution containing polymerized polyphenols for one week [94]. A significantly lower DMFT score was also observed in 14 year old children who drank tea (whether with added sugar or not) in comparison to coffee drinkers [95].

Zhang and Kashket [96] reported, moreover, that green tea extracts inhibit human salivary amylase and may reduce the cariogenic potential of starch-containing food such as crackers and cakes because they may reduce the tendency of this kind of food to serve as slow-release sources of fermentable carbohydrate.

The possible protective effect of cocoa on dental caries is also receiving increasing attention, but previously published data concerning the anticariogenic effects of constituents of chocolate are conflicting. An early study indicated that a high-sucrose diet was equally cariogenic in the presence or absence of cocoa bean ash [97], while the incorporation of cocoa powder or chocolate into hamster diets was reported to reduce caries [98]. Another *in vivo* study showed that the cariogenic potential indices (CPI) of chocolate with high cocoa levels was less than 40% that of sucrose (10% w/v) and also lower than that of chocolate containing low cocoa levels [99]. The anticariogenic effects of polyphenols isolated from cocoa have not yet been studied. Recently, the ground husks of cocoa beans, which are a product of cocoa manufacturing that have a high polyphenol content, were used to prepare a mouthwash for children. The regular use of this mouthwash gave a 20.9% reduction in mutans streptococci counts and was even more effective in decreasing plaque scores [100].

## 5. Conclusions

The studies carried out in recent decades have confirmed the antibacterial role of polyphenols: they may reduce bacterial growth rate and adherence to tooth surface, and also can perform inhibitory effects on the enzymatic activity of glucosyltransferase and amylase. Moreover, polyphenols largely occur in flowering plants and could be used at a reasonable cost in the preparation of specific remedies. Flavonoids seem to be particularly promising anticariogenic molecules, but research on the relationships between chemical structure and anti-microbial activity of these compounds, as well as their synergistic/antagonistic effects, is still required.
